# The core of maintaining neuropathic pain: Crosstalk between glial cells and neurons (neural cell crosstalk at spinal cord)

**DOI:** 10.1002/brb3.2868

**Published:** 2023-01-05

**Authors:** Tianrui Zhang, Mingqian Zhang, Shuang Cui, Wulin Liang, Zhanhong Jia, Fanfan Guo, Wenjing Ou, Yonghong Wu, Shuofeng Zhang

**Affiliations:** ^1^ Department of Pharmacology of Traditional Chinese Medicine Beijing University of Chinese Medicine Beijing China

**Keywords:** astrocytes, crosstalk, hyperalgesia, microglia, neuron, neuropathic pain

## Abstract

**Background:**

Neuropathic pain (NP) caused by the injury or dysfunction of the nervous system is a chronic pain state accompanied by hyperalgesia, and the available clinical treatment is relatively scarce. Hyperalgesia mediated by pro‐inflammatory factors and chemokines plays an important role in the occurrence and maintenance of NP.

**Data treatment:**

Therefore, we conducted a systematic literature review of experimental NP (PubMed Medline), in order to find the mechanism of inducing central sensitization and explore the intervention methods of hyperalgesia caused by real or simulated injury.

**Result:**

In this review, we sorted out the activation pathways of microglia, astrocytes and neurons, and the process of crosstalk among them. It was found that in NP, the microglia P2X4 receptor is the key target, which can activate the mitogen‐activated protein kinase pathway inward and then activate astrocytes and outwardly activate neuronal tropomyosin receptor kinase B receptor to activate neurons. At the same time, activated neurons continue to maintain the activation of astrocytes and microglia through chemokines on CXCL13/CXCR5 and CX3CL1/CX3CR1. This crosstalk process is the key to maintaining NP.

**Conclusion:**

We summarize the further research on crosstalk among neurons, microglia, and astrocytes in the central nervous system, elaborate the ways and connections of relevant crosstalk, and find potential crosstalk targets, which provides a reference for drug development and preclinical research.

## Graphical abstract

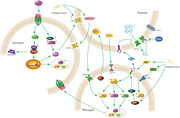

## 1 INTRODUCTION

Neuropathic pain (NP) is an injury or disease of the somatosensory nervous system, characterized by persistent spontaneous pain, hyperalgesia, and allodynia (Sumizono et al., 2018). Hyperalgesia is usually characterized by increased pain caused by noxious stimuli, whereas allodynia is characterized by pain caused by non‐noxious stimuli (Jensen & Finnerup, 2014; Yekkirala et al., 2017). NP‐induced hyperalgesia is a remodeling mechanism of peripheral and central nociceptors. Continuous stimulation, such as tissue or nerve injury, will reshape nociceptors to produce hyperalgesia. In particular, peripheral sensitization, including abnormal excitation of peripheral nociceptors and dorsal root ganglion (DRG), is also a sign of primary hyperalgesia (Woolf & Ma, 2007). Hyperalgesia begins with peripheral nerve sensitization and finally forms a series of neural plasticity changes in spinal cord and brain, which is called central sensitization (CS) (Deval et al., 2008). CS, which is secondary hyperalgesia, including the imbalance of ascending excitation pathway and descending pain regulation system, plays an important role in the development and maintenance of chronic pain (Boadas‐Vaello et al., 2016). In the ascending excitation pathway, the amplification of nerve signals in the central nervous system (CNS) produces a state of hyperactivity and overexcitation of neurons in the spinal cord and brain, leading to hyperalgesia (Woolf, 2011). Descending pain regulation system mostly originates from the superior part of spinal cord, including periaqueductal gray (PAG) of midbrain, gray matter is indirectly projected to the spinal cord through the rostral ventromedial medulla (RVM), lateral and caudal dorsal reticular nuclei and ventrolateral medulla, PAG‐RVM system is an important regulatory center and also a research hotspot (Heinricher et al., 2009). PAG receives signals from frontal cortex, amygdala and hypothalamus and projects the input to RVM and lower brainstem, which affects pain perception (Zhang & Lee, 2018). PAG integrates these signals and transmits them to RVM, which can promote and/or inhibit pain according to specific activation pathways (Kwon et al., 2014).

NP is often accompanied by a CS of the spinal cord. CS at the spinal level occurs in the dorsal horn of the spinal cord, resulting in enlarged receptive field of interneurons and enhanced pain sensitivity. The spinal dorsal horn (SDH) is the site between the peripheral sensory neurons and the secondary interneurons of the spinal cord (Foster et al., 2015). The two main stages are as follows: (1) The glutamate release from primary afferent neurons is associated with the acute phase mediated by *N*‐methyl‐d‐aspartate receptor (NMDAR) on SDH postsynaptic neurons; (2) chronic phase mediated by activation of spinal microglia and transcription of pain regulatory peptide (Latremoliere & Woolf, 2009). After spinal cord nerve or tissue injury, chemokines sensitize peripheral and central nerves (Ji et al., 2014). The increase of inflammatory mediators can cause afferent nerve hypersensitivity and spontaneous pain by changing the expression of voltage‐gated sodium, calcium, and potassium channels (Abdulla & Smith, 2001; Alles & Smith, 2018). Peripheral sensitization of DRG and CS of spinal cord and superior spinal cord are still the main mechanisms and research hotspots of NP (Ji et al., 2014). CS of the supraspinal central region, as in the spinal cord, revealed an increase in vesicular glutamate transporter 2 (Vglut2) in the thalamus, PAG, and amygdala in a model of NP (Wang et al., 2015). In the chronic constriction injury (CCI) model, glial cell activation promotes a downstream facilitation of NP by PAG (Ni et al., 2016). The anterior cingulate cortex (ACC) of NP mice shows an increased expression of the astrocyte marker glial fibrillary acidic protein (GFAP) (Masocha, 2015). In addition to its role in learning and memory, the hippocampus and/or amygdala may also play a role in chronic pain and be influenced by pain‐induced plasticity (McCarberg & Peppin, 2019). Inflammatory cytokines modulate synaptic plasticity in a region‐dependent manner, that is, enhancing synaptic connections in the dorsal horn of the spinal cord and reducing synaptic connections in the hippocampus, respectively, leading to persistent pain and memory/emotion deficits (Liu, 2022). Overproduction of pro‐inflammatory cytokines and glial cell activation impairs long‐term potentiation (LTP) in the hippocampus and reduces excitatory synapses in the hippocampus (Cowley et al., 2012; Pickering & O'Connor, 2007). Specifically, hippocampal network activity is associated with increased pain, whereas individual sensitivity to NP expectations is associated with differential sensitization of the hippocampus and amygdala (Ziv et al., 2010).

Since the beginning of the 21st century, Hashizume et al. (2000) have put forward the hypothesis that the central immune mechanism is an important factor in the development and maintenance of chronic NP. They proved that the activation of spinal cord microglia and the increase of interleukin 1β (IL‐1β) are involved in the process of NP through the models of lumbar radiculopathy. This study has changed people's perception of NP. In recent years, with in‐depth research, most scholars believe that crosstalk between nonneuronal cells play an important role in the induction and maintenance of NP in addition to CS mediated by persistent neuronal excitation (Ji et al., 2016). Astrocytes, oligodendrocytes, and microglia are the main types of glia in mammalian CNS (Jha et al., 2019). In the mature CNS environment, each nonneuronal cell has a unique function of supporting neurons: Astrocytes maintain the homeostasis of ions and neurotransmitters, improve synaptic connections, and ensure the normal metabolism of neurons; microglia can monitor synaptic elements and networks, induce or remove synaptic structures, and regulate neuronal activity; oligodendrocytes form myelin sheath to protect and nourish the myelinated ganglion (Matejuk & Ransohoff, 2020). Peripheral nerve injury, inflammation, and other noxious stimuli can induce the sequential activation of microglia and astrocytes and then affect the continuous activation of neurons (Zhang et al., 2017). It is of great significance to explore the information exchange between glial cells and neurons in chronic pain for understanding the mechanism of pain sensitization and finding new drug targets.

## 2 CYTOKINE‐MEDIATED NEUROPATHIC PAIN

It is well known that inflammatory mediators promote angiogenesis and interact with sensory neurons that induce pain signals. Peripheral nerve injury induces macrophage aggregation and cytokine production and leads to sensitization and inflammatory response of local neurons in the spinal cord (Malcangio, 2019). Under the action of cytokines, it can also increase the discharge of spinal cord neurons and further aggravate the generation of pain (Xanthos & Sandkuhler, 2014). In Table 1, we summarize the effects of some cytokines on spinal cord neurons. Different cytokines often activate neurons through different pathways. In this section, we outline how important cytokines participate in and maintain NP.

**TABLE 1:** Related cytokines for neuron activation

Cytokines	Receptor	Effect result
IL‐1β	IL‐1R	Activate TRPV1; activate NMDA receptor; phosphorylate Nav1.8
IL‐5	IL‐5R	Induce neuronal sensitization
IL‐6	gp130	Activate TRPA1 and TRPV1; induce neuronal sensitization
IL‐17A	IL‐17AR	Induce neuronal sensitization
TNF‐α	TNFR1	
TNFR2	Phosphorylate Nav1.8, Nav1.9; activate AMPA, NMDA; inhibit GABA	
PGE2	EP1‐4	Induce neuronal sensitization
5‐HT	5‐HT2	Induce neuronal sensitization
His	H1/2	Phosphorylated Nav1.8
NGF	TrkA	Activate TRPV1, phosphorylate Nav1.7
BDNF	TrkB	Downregulation inhibits KCC2

Abbreviation: AMPA, α‐amino‐3‐hydroxy‐5‐methyl‐4‐isoxazole propionic acid; BDNF, brain‐derived neurotrophic factor; GABA, γ‐aminobutyric acid; IL, interleukin; NMDA, *N*‐methyl‐d‐aspartate; TNF, tumor necrosis factor; TNFR, tumor necrosis factor receptor; TrkB, tropomyosin receptor kinase B.

### 2.1 Neuronal activation and hyperalgesia

After peripheral nerve injury, the persistent activation of interneurons in SDH mediates the process of hyperalgesia. In addition to the activation of interneurons induced by the stimulation of sensory neurons, the activation of interneurons depends more on cytokines secreted by immune cells (Verri et al., 2006). The role of cytokines in pain can be confirmed in a variety of pain models, such as arthritis, rheumatoid arthritis, and NP. Among them, IL‐1β, interleukin 5 (IL‐5), interleukin 6 (IL‐6), tumor necrosis factor α (TNF‐α), interleukin‐17A (IL‐17A) has been shown to act directly on nociceptive neurons (Pinho‐Ribeiro et al., 2017). In neurons, specific intracellular signal transduction pathways, such as cytokine, lipid, and growth factor receptors, can lead to the phosphorylation of ion channels Nav1.7, Nav1.8, Nav1.9, TRPV1, and TRPA1, resulting in the generation of action potential and the deepening of pain sensitization (Pinho‐Ribeiro et al., 2017). See Table 1 for details.

### 2.2 Effects of inflammatory factors on neuronal activation

IL‐1β has been shown to affect the Ca^2+^ conduction of NMDARs on neurons in a dose‐dependent manner. It can inhibit and improve Ca^2+^ influx at high and low concentrations, respectively, and then trigger the intracellular signal cascade required for synaptic plasticity (Rizzo et al., 2018). IL‐1β inducing P38 mitogen‐activated protein kinase (MAPK) phosphorylation by activating Nav1.8 sodium channel sensitizes nociceptive neurons, resulting in increased action potential production, and activates IL‐1 receptor on nociceptive neurons to increase the expression of capsaicin receptor (TRPV1), resulting in mechanical and thermal hyperalgesia (Binshtok et al., 2008; Ebbinghaus et al., 2012). Similarly, IL‐1β was injected into IL‐1 receptor knockout mice, the mice did not show any mechanical or thermal hyperalgesia after injection, which further confirmed that the IL‐1β is dependent on IL‐1 receptor to induce hyperalgesia (Mailhot et al., 2020).

The regulation of neurons in TNF‐α mainly depends on tumor necrosis factor receptor (TNFR)1 and TNFR2, which can regulate different nociceptors (Spicarova et al., 2011). TNF‐α‐induced neuronal sensitization and hyperalgesia are dependent on TRPV1 and TRPA1 (Fernandes et al., 2011). Hyperalgesia experiments in mice knocked out of TNFR1 or TNFR2 by transgenic technology found that TNFR1 is related to the development of thermal hyperalgesia, whereas mechanical allodynia is related to TNFR1 and TNFR2 (Steeland et al., 2018). TNF‐α also changes neuronal excitability through P38 MAPK‐mediated phosphorylation of Nav1.8 and Nav1.9 sodium channels, thereby inducing the rapid regulation of nociceptor sensitivity (Gudes et al., 2015). TNF‐α increases the frequency of neuronal spontaneous/evoked potentials, as well as the α‐amino‐3‐hydroxy‐5‐methyl‐4‐isoxazole propionic acid receptor (AMPA) and NMDA‐induced currents in SDH neurons, by inhibiting membrane surface γ‐aminobutyric acid (GABA) can promote excitement of dorsal horn neurons (Spicarova et al., 2011). In addition, TNF‐α can enhance excitatory synaptic transmission and inhibit inhibitory synaptic transmission of SDH II lamellar neurons (Kawasaki et al., 2008).

IL‐6 is a common and typical pro‐inflammatory factor, which mediates biological effects through a hexamer complex composed of IL‐6 itself, its receptor IL‐6R and glycoprotein 130 (gp130) (Kaur et al., 2020). This complex can activate a variety of signaling mechanisms to perform various biochemical functions. In the CCI pain model, it was found that the expression of IL‐6Ra was significantly raised in SDH nociceptive neurons (Gui et al., 2020). Similarly, soluble gp130 inhibited IL‐6 signal transduction in spinal cord neurons and attenuated the hyperactivity of spinal cord neurons in peripheral inflammation (Vazquez et al., 2012). In addition, TNF‐α can also induce the hyperexcitability of spinal cord neurons through IL‐6/sIL‐6R and then induce pain sensitization (Konig et al., 2016).

In the process of peripheral injury, the inflammatory factors produced by microglia and astrocytes can directly act on the receptors or ion channels of nociceptor neurons and then induce sustained excitation of neurons, leading to pain sensitization.

## 3 MICROGLIA AND NEUROPATHIC PAIN

Microglia exist in a resting state under normal physiological conditions. When the external environment changes dramatically, microglia are activated rapidly (Tu et al., 2021). Activated microglia show hypertrophy, proliferation, and cell body thickening, which is called activation (Savage et al., 2019). Studies have shown that microglia are immune active cells in the CNS, which regulate the transmission of excitation in SDH. Peripheral nerve injury can directly induce the activation of microglia in SDH (Inoue & Tsuda, 2018). Activated microglia show significant changes in the expression of various genes, including cell surface receptors (such as purinergic receptor P2X) and intracellular signaling molecules (such as MAPKs) for neurotransmission, and bioactive diffusing factors (such as pro‐inflammatory cytokines and neurotrophic factors) (Tsuda et al., 2003). It has been reported that minocycline (a microglia inhibitor) can effectively inhibit the activation of neurons and block pain sensitization (Asano et al., 2020). In this section, we summarize the types and ways of microglia polarization and how microglia activate, participate in and maintain NP.

### 3.1 Microglia polarization

**FIGURE 1:**
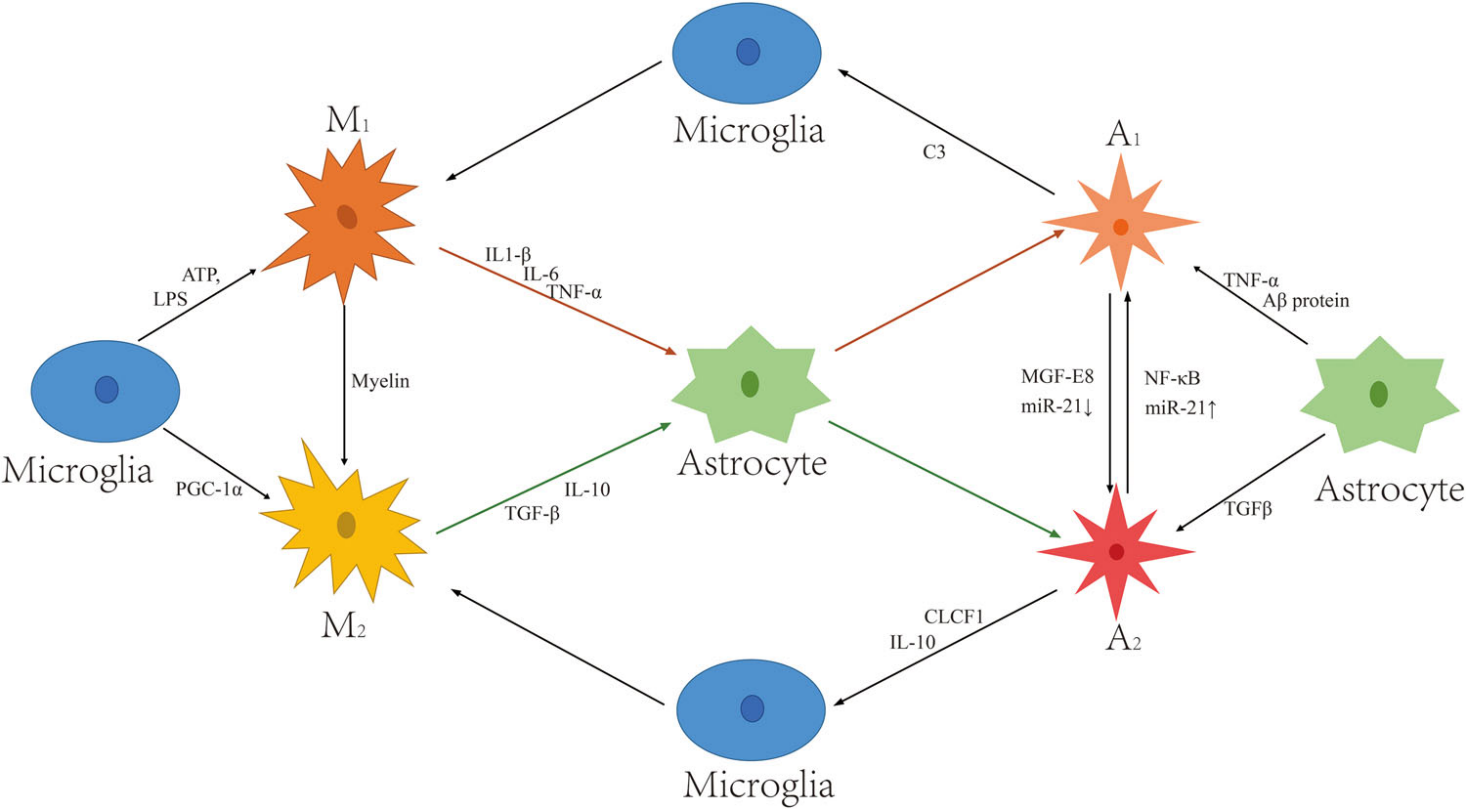
Polarization and crosstalk of microglia and astrocytes. Activation, polarization, and transformation of microglia and astrocytes are regulated by relevant cytokines. Under the induction of adenosine triphosphate (ATP) or LPS, resting M0 microglia can be polarized to M1 microglia, and under the induction of PGC‐1α, can be polarized to M2 microglia. M1 microglia can be transformed into M2 microglia under the induction of myelin. Resting A0 astrocytes can polarize into A1 astrocytes upon the induction of tumor necrosis factor α (TNF‐α) or Aβ protein and into A2 astrocytes upon the induction of transforming growth factor β (TGF‐β). A1 astrocytes can be transformed into A2 astrocytes in the presence of MGF‐F8 or downregulated miR‐21; A2 astrocytes can be transformed into A1 astrocytes in the presence of NF‐κB pathway or upregulated miR‐21. M1 microglia can directly induce the polarization of A0 astrocytes to A1 astrocytes by releasing interleukin (IL)‐1β, IL‐6, and TNF‐α. M2 microglia can directly induce the polarization of A0 astrocytes to A2 astrocytes by releasing IL‐10, TGF‐β. A1 astrocytes induce M0 microglia to polarize to M1 type by releasing C3. A2 astrocytes directly induce M0 microglia to polarize to M2 type by releasing CLCF1 or IL‐10.

The classical activation of microglia is related to the production of pro‐inflammatory factors. According to the difference of secreted factors, the phenotype of microglia is the classical activated phenotype M1 or the representative type M2, which are involved in tissue injury and tissue repair, respectively (Feng et al., 2019). The existence of M1/M2 pattern provides a new research idea for the activation of microglia in CNS diseases (Tang & Le, 2016). M1 exists in a pro‐inflammatory state, leading to the release of inflammatory factors. In addition to inducing the production of nitric oxide synthase (iNOS), it also activates the release of pro‐inflammatory cytokines, such as interleukins IL‐1β, IL‐6, and TNF‐α (Jiang et al., 2020). Type M1 microglia can be distinguished by surface antibodies, commonly including integrin α‐M (CD11b), which increased the expression of differentiation marker cluster 86 and 16/32 (CD86, CD16/32) (Jiang et al., 2020). M2 exists in an anti‐inflammatory state, secretes anti‐inflammatory factors and neurotrophic factors, and further promotes the repair of the nervous system (Zhang et al., 2018). Further subdivided into M2a, M2b, and M2c, the expression markers in rodents include anti‐inflammatory cytokines, arginase‐1 (arg1), transforming growth factor β1 (TGF‐β1) CD206 and chitinase‐3‐like protein‐3 (Ym1) (Yang et al., 2017). Upward peroxisome proliferator‐activated receptor‐γ coactivator‐1α(PGC‐1α) can effectively inhibit the expression of inflammatory factors in microglia and promote the transformation of microglia to M2 type (Yang et al., 2017). Myelin was engulfed by M1 microglia in vitro, which induced the transition from M1 polarization to M2 polarization (Du et al., 2017). See Figure 1 for details.

### 3.2 Microglia activation and related pathways

The P2 purinergic receptor family on the surface of microglia is the key to the activation of microglia (Agostinho et al., 2020). The P2 purinergic receptor family includes ion channel receptors P2X and metabotropic G protein‐coupled receptors P2Y. The subtypes are P2X1–7 and P2Y1, 2, 4, 6, 11, 12, 13, and 14 (Tsuda et al., 2013). Among them, P2X4 and P2X7 in the P2X family allow organic macromolecules to pass between cells, which can lead to hyperalgesia (Bernier et al., 2012). P2X receptor family exists in many tissues and participates in many functions, including synaptic transmission, muscle contraction, platelet aggregation, inflammation, neuropathic, and inflammatory pain (Bernier et al., 2018). As one of the most sensitive receptors in the P2X family, P2X4 is widely present in peripheral and central neurons and microglia. It is involved in mediating NP and the maintenance of hyperalgesia (Suurvali et al., 2017). Tyrosine kinase, a member of Src family kinases, is one of the important molecules of fibronectin/integrin signal transduction in microglia and can participate in the regulation of P2X4 receptor expression (Matsumura et al., 2016). P2X4 knockout mice did not show pain behavior after nerve injury; for wild‐type mice, blocking the P2X4 receptor with antagonist can also inhibit the pain behavior caused by the herpes virus HSV‐1 (Matsumura et al., 2016; Ulmann et al., 2008).

Interferon regulatory factor 8 (IRF8) is a member of the IRF family (IRF1–9), which is expressed in immune cells, such as lymphocytes and dendrites. Masuda et al. (2012) recently found that the expression of IRF8 was selectively upregulated in microglia after nerve injury in the spinal cord. At the same time, IRF8‐deficient mice showed a decrease in tactile allodynia induced by nerve injury. Masuda et al. (2014) also found that IRF5 was the target of IRF8. After nerve injury, ipsilateral SDH microglia also selectively expressed IRF5. Fibronectin in activated microglia regulates the transfer of IRF5 from the cytoplasm to the nucleus, and IRF5 induces the expression of P2X4 receptor by directly binding to the promoter region of the P2rX4 gene (Matsumura et al., 2016). Therefore, the IRF8–5 transcription axis contributes to the activation of P2X4 receptors on the surface of ipsilateral SDH glial cells after nerve injury.

Gangliosides located in the extramembranous lobules are components of lipid rafts, which are related to the communication between nerve and extracellular microenvironment or contribute to the interaction between neuron and glial cells. Lim et al. (2020) found that after peripheral nerve injury, ganglioside (GT1b) in sensory neurons was transported to the spinal cord through axons and released in the spinal cord. In the spinal cord, GT1b acts as an endogenous agonist of toll like receptor 2 of microglia, activates the P38 MAPK pathway, and induces microglia to secrete IL‐1β, TNF‐α, brain‐derived neurotrophic factor (BDNF) and nitric oxide (NO) production, which in turn leads to CS (Lim et al., 2020; Liu et al., 2017).

MAPKs are evolutionarily conserved intracellular signal molecules serine/threonine protein kinases, which play a key role in regulating gene expression, promoting disease development and pain maintenance (Mai et al., 2020). The MAPK family is divided into three main members: extracellular signal‐regulated kinase (ERK), c‐Jun N‐terminal kinase (JNK), and P38 MAPK (Kumar et al., 2003). P38 phosphorylation of microglia is a common pathway for activation of cell surface receptors on microglia (Kumar et al., 2003). Many purine receptors can be used as promoters and regulators of MAPK pathway to induce hyperalgesia (Burnstock, 2009); taking P2X7 and P2Y12 as examples, the upregulation of both receptors can activate P38 MAPK signal and participate in the induction of NP caused by spinal nerve ligation (Li et al., 2017; Yu et al., 2019). When activated, P38 MAPK increases the synthesis and release of many microglia mediators, such as TNF‐α, IL‐1β, IL‐6, PGE2, and promotes the development of NP (Ji et al., 2013). In addition to P38 MAPK, the phosphorylation of ERK 1/2 and 5 (ERK1/2 and ERK5) was also found in spinal microglia after nerve injury. Both ERK1/2 and ERK5 regulate NP (Chen et al., 2018). The expression of ERK1/2 is rare under normal healthy conditions or harmless stimuli. In animal models of pain, the upregulation of ERK1/2 mainly exists in the superficial layer of SDH (Borges et al., 2015). The activation of ERK5 mainly occurs in microglia. After activation, it can mediate the transmission of nociceptive signals between microglia and neurons, helping to form and maintain pain hyperalgesia or allodynia (Sun et al., 2013).

## 4 ASTROCYTES AND NEUROPATHIC PAIN

Compared with microglia, astrocytes are activated more persistently under chronic pain conditions, indicating that they have a greater contribution to hyperalgesia (Ji et al., 2016). Astrocytes are the most abundant cell type in the CNS. After harmful stimulation or nerve damage, the phenotype, function, and gene expression of astrocytes will undergo significant changes, which called reactive astrocytes proliferation (Hara et al., 2017). In this section, we summarize the types and ways of astrocyte polarization and how astrocytes activate, participate in, and maintain NP.

### 4.1 Astrocyte polarization

At present, some scholars have proposed that nerve injury can induce two different types of reactive astrocytes, called A1 reactive astrocytes and A2 reactive astrocytes (Liddelow et al., 2017). A1 astrocytes can secrete a class of soluble toxins, which induce the rapid death of neurons and mature oligodendrocytes (Miller, 2018). Neuroprotective A2 astrocytes have been shown to secrete a variety of neurotrophic factors, which can promote nerves cell survival and axon formation/repair (Diniz et al., 2014; Liddelow & Barres, 2017). Activated microglia induce the transformation of naive astrocytes into A1 astrocytes by releasing IL‐1 and TNF‐α (Liddelow et al., 2017). Preventing A1 formation, promoting A1 reversal or blocking A1 neurotoxin has great potential for reducing neuronal loss. In contrast, the activation of A2 astrocytes is induced by ischemia, whereas A2 astrocytes promote the expression of TGF‐β, which strongly promote neuron survival and tissue repair (Xu et al., 2018). Therefore, it is particularly important to explore the transformation of astrocytes A1 and A2 in chronic NP. See Figure 1 for details.

### 4.2 Astrocyte activation and related pathways

Astrocytes are usually identified by immunohistochemistry to determine the presence of glial fibers, and their main component is GFAP (Old et al., 2015). Activated astrocytes may promote the development and maintenance of chronic pain by releasing molecular signals. The proliferation of astrocytes has been found in various NP models such as radiculectomy and spinal nerve ligation (Ji et al., 2013). Astrocytes can be activated through a variety of ways. Activated astrocytes express many ligands, such as excitatory amino acids, substance P, calcitonin gene‐related peptide, and others, which can regulate the upregulated receptors of SDH neurons after peripheral nerve injury (Durkee & Araque, 2019). It is worth noting that astrocytes also have receptors for a variety of cytokines and chemokines, which can be activated by ligands released by microglia, such as TNF‐α and CCL2 (Gruber‐Schoffnegger et al., 2013). It has been pointed out in the literature that intrathecal injection of TNF‐α in mice activates astrocytes and induces mechanical allodynia in uninjured mice (Gao et al., 2010). Interestingly, intrathecal injection of fluorocitrate, an inhibitor of astrocytes, can effectively inhibit paclitaxel induced NP in rats, which can also prove that astrocytes participate in the process of pain sensitization (Xu et al., 2016).

Complement3 (C3) and S100A10, a member of the S100 calcium binding protein family, are specific markers for A1 and A2 astrocytes, respectively (Fujita et al., 2018; Liddelow et al., 2017). Nuclear transcription factor (NF‐κB) signaling pathway plays an important role in physiological and pathological processes, such as immune response, cell proliferation, and cell apoptosis. When the inducing factor works, IκBα is degraded, thereby promoting the phosphorylation of NF‐κB, making NF‐κB transfer from the cytoplasm to the nucleus, thereby activating gene expression. NF‐κB is also involved in astrocyte‐mediated neuroinflammatory response. In the Aβ42‐induced A1‐type astrocyte activation experiment, it was also found that the content of p‐IκBa and p‐NF‐κB increased (Gabel et al., 2016; Xu et al., 2018). Mice knocking out the NF‐κB gene in astrocytes can be greatly improved in the spinal cord injury model, and the expression of chemokines CXCL10 and CCL2 and the cytokine TGF‐β in the spinal cord are also significantly downregulated (Brambilla et al., 2005). Signal transducer and activator of transcription 3 (STAT3) play an important role in the polarization of astrocytes (Cappoli et al., 2020). Recent studies have shown that the activation of type A1 astrocytes is regulated by the phosphorylation of STAT3 (Qian et al., 2019). Researchers found that milk fat globule epidermal growth factor 8 (MFG‐E8) inhibits the expression of A1 astrocyte marker C3 in vitro to affect astrocyte activation and also found that TGF‐β is expressed in A2 astrocytes enhanced in the process. The use of anti‐MFG‐E8 antibody and NF‐κB inhibitor can relieve the transformation inhibition of A1 astrocytes. This result reveals for the first time the regulatory effect of MFG‐E8 on the changes of A1/A2 astrocytes through the NF‐κB pathway (Xu et al., 2018). In addition, miR‐21, a small RNA molecule, can participate in the regulation of the polarization of astrocytes. The downregulation of miR‐21 can induce the transformation of A1 astrocytes into A2 astrocytes; when miR‐21 is upregulated, the result was opposite (Su et al., 2019). See Figure 1 for details.

## 5 THE CROSSTALK OF MICROGLIA, ASTROCYTES, AND NEURONS INDUCES NEUROPATHIC PAIN

In the SDH, activated microglia are very important in causing neural plasticity and NP. Even without harmful stimulation, microglia contribute to the generation and maintenance of pain and affect the activation of astrocytes (Ho et al., 2020). In this section, we outline microglia‐induced neuronal activation and its effects on astrocytes. At the same time, we describe the mechanism of neurons and astrocytes in maintaining the activated state of microglia and how they participate in NP.

### 5.1 Crosstalk between microglia and neurons induces hyperalgesia

The extracellular adenosine triphosphate (ATP) released when SDH neurons are injured is also involved in activating microglia, causing NP (Masuda et al., 2016). ATP‐induced activation of the P2X4 receptor in microglia leads to the release of BDNF, which acts on the tropomyosin receptor kinase B receptor of SDH neurons and then downregulates the K^+^/Cl^−^ cotransporter 2 (KCC2) (Lee‐Hotta et al., 2019; Malcangio, 2017). KCC2 is a channel protein that simultaneously discharges K^+^ and Cl^−^ in neurons to the outside of the membrane. It can regulate neuronal excitability by adjusting the concentration of Cl^−^ intracellular and extracellular and the inhibitory effect of GABAergic nerves (Wilke et al., 2020). Downregulation of KCC2 results in the inhibition of Cl^−^ efflux in neurons. On the one hand, it reduces the Cl^−^ concentration difference inside and outside the cell, which leads to the decrease of Cl^−^ inward driving force under the action of GABAergic action, the obstruction of membrane potential hyperpolarization process, and the weakening of nerve excitation inhibition. On the other hand, it leads to the accumulation of intracellular Cl^−^ and increases the outward driving force of Cl^−^, which leads to the downregulation of GABA and depolarization, then the transformation from inhibitory effect to excitatory effect, showing the effect of enhancing the transmission of injury regulation (Akita & Fukuda, 2020; Malcangio, 2019). P2X4 receptor activation leads to the release of BDNF from microglia as a key factor in sending signals to the first layer of neurons, leading to increased neuronal excitability, which in turn leads to CS. By enhancing KCC2 activity and central penetration of monoclonal antibodies or intrathecal delivery of small molecule antagonists to block P2X4 to regulate the P2X4/BDNF microglia pathway, it has shown a significant antinociceptive effect in NP models, which is also drug intervention that provides new ideas for NP (Williams et al., 2019).

Chemokines are small molecular proteins (10–20 kDa), which have a regulatory effect on all immune cells, including movement, distribution, development, and regulation of homeostasis (Griffith et al., 2014). Neurons can express a variety of chemokines as communication molecules between neurons and nonneuronal cells. As the expression of neuronal chemokines is significantly upregulated under neuronal stress or injury, these neuronal chemokines may transmit dangerous signals from damaged neurons. Fractal protein (Fractalkine, FKN, CX3CL1) is a member of the cytokine chemokine family and the only member of the CX3C class of chemokines (Clark et al., 2011). In the spinal cord, CX3CL1 is expressed in neurons (Clark et al., 2009). CX3CR1 is the only receptor for the chemokine CX3CL1, which is specifically expressed on the microglia of the CNS (Zhou et al., 2020). The unconnected domain of CX3CL1 transmits signals to microglia through CX3CR1.

The chemokine pair CX3CL1/CX3CR1 has been shown to participate in NP through neuron–microglia interactions in the spinal cord (Zhang et al., 2017). Cathepsin S (Cat S) is expressed in vascular smooth muscle cells and can cut CX3CL1 into a soluble smaller form (50–55 kDa) (Fonovic et al., 2013). In the spinal cord, Cat S is secreted by the activated microglia in the spinal cord and is upregulated after nerve injury. It cleaves soluble CX3CL1 from local neurons and primary afferent nerve fibers in the spinal cord and then acts on CX3CR1 (Malcangio, 2019). P38 is a member of MAPKs and an important downstream kinase of CX3CL1/CX3CR1 signal transduction (Clark et al., 2007). Intrathecal injection of CX3CR1 neutralizing antibody can reduce the activation of spinal microglia after nerve injury; in contrast, intrathecal injection of CX3CL1 induces the P38 activation of microglia (Zhuang et al., 2007). The activation of the P38 pathway eventually induces microglia to synthesize pro‐inflammatory cytokines, such as TNF‐α, IL‐1β, and IL‐6. These cytokines diffuse to the neuronal receptors of SDH, leading to spontaneous discharge and hypersensitivity reactions specific to CS, and provide a feedback mechanism through which to further maintain the activated state of microglia. Interestingly, Biber pointed out that the endogenous expression of CX3CL1 in neurons can limit the activation of CX3CR1 on microglia, thereby forcing microglia to remain quiescent (Biber et al., 2007; Pawelec et al., 2020). It can be seen that the effect of CX3CL1/CX3CR1 chemokines on neurons and microglia is controversial, and it is worthy of in‐depth study.

**FIGURE 2:**
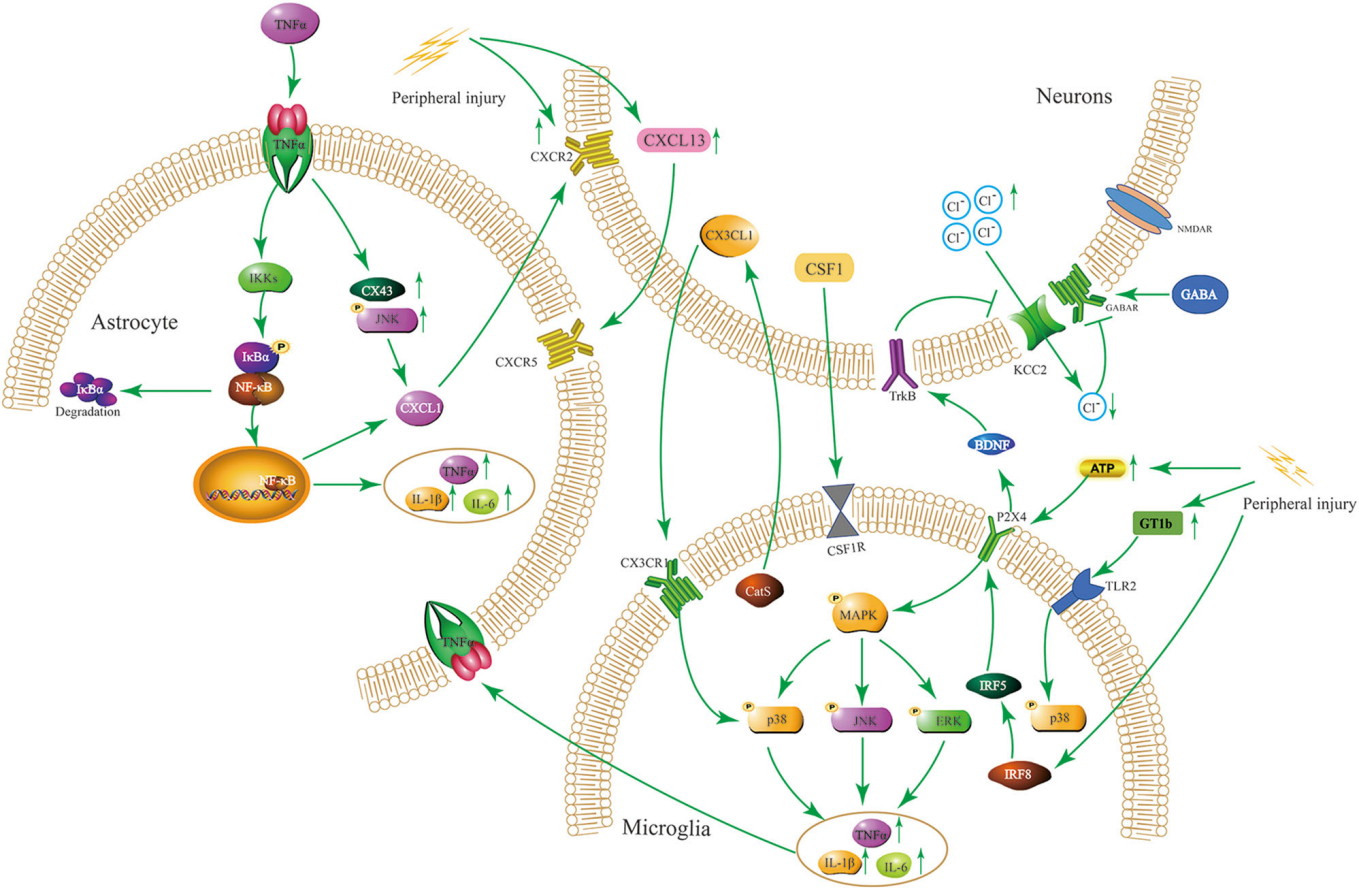
Microglia–astrocyte–neuron crosstalk diagram. Under peripheral nerve injury, microglia activate the surface receptor P2X4 under the action of adenosine triphosphate (ATP), secrete BNDF extracellularly to act on neurons, and then activate the MAPK inflammatory pathway, secrete interleukin (IL)‐1β, IL‐6, tumor necrosis factor α (TNF‐α) and other inflammations; by upregulating GT1b, it acts on toll like receptor 2 (TLR2) and activates the P38 MAPK pathway; it can also directly upregulate Interferon regulatory factor (IRF)8 and IRF5 in microglia to continue to activate P2X4 receptors under peripheral nerve injury. TNF‐α acts on the TNF‐α receptor of astrocytes, activates the typical NF‐κB pathway, and then secretes related inflammatory factors; TNF‐α receptors act on neuronal CXCR2 receptors by upregulating CX43, c‐Jun N‐terminal kinase (JNK) and then secreting chemokine CXCL1. Neurons upregulate the chemokine CXCL13 under the influence of peripheral nerve injury and act on the CXCR5 receptor of astrocytes; Cat S secreted by activated microglia can directly regulate the intraneuronal chemokine CX3CL1 and act on the CX3CR1 receptor of microglia to continuously activate microglia; activated neurons secrete CSF‐1, which can act on the CSF‐1 receptor of microglia and still maintain the activated state of microglia; brain‐derived neurotrophic factor (BDNF) acts on the neuron tropomyosin receptor kinase B (TrkB) receptor to inhibit the activity of the KCC2 transporter, so that the intracellular Cl^−^ concentration is reduced, the GABAergic nervous system is inhibited, and the neuron is continuously excited.

In addition, colony stimulating factor 1 (CSF‐1) was significantly upregulated in damaged sensory neurons (Suter, 2016). After CSF‐1 is transported to the central end of nociceptors in the spinal cord, it is released and attached to its microglia CSF‐1 receptor, and participates in the proliferation and activation of microglia in the spinal cord (Guan et al., 2016). The CSF‐1 receptor is upregulated in activated spinal microglia. Neuron CSF‐1 (or exogenous CSF‐1) induces the activation marker genes of spinal microglia in a DAP12‐dependent manner. DAP12 stimulates microglia proliferation in an independent manner (Tozaki‐Saitoh & Tsuda, 2019). Conditional CSF‐1 knockout in sensory neurons prevents the development of mechanical hyperalgesia, and the inhibition of CSF‐1R kinase activity can reduce mechanical hyperalgesia (Lee et al., 2018). See Figure 2 for details.

Spinal LTP is an important form of spinal synaptic plasticity that contributes to CS and pathological pain (Liu & Zhou, 2015). Activation of spinal microglia is sufficient and necessary for the induction of spinal LTP at C‐fiber synapses of layer I neurons (Chen et al., 2018). The mechanism may involve the release of ATP, P2X4 or P2X7 receptors, P38 phosphorylation, and IL‐1 in microglia (Zhuo et al., 2011). Li et al. activated microglia in the dorsal horn of the spinal cord and induced LTP and nociceptive hypersensitivity by electrical stimulation (10 V, 5 Hz, and 10 s) without damaging nerve fibers and successfully induced crosstalk between microglia and neurons using BDNF to trigger spinal LTP and CS (Zhou et al., 2019). Activated microglia release IL‐1β and regulate NMDARs in postsynaptic neurons, ultimately enhancing the release of presynaptic neurotransmitters and inducing neuronal LTP (Clark et al., 2015). Gliogenic LTP, as well as other forms of diffuse and glial‐mediated CS, may underlie widespread pain in patients with NP, particularly in patients with superimposed chronic pain conditions (Ji et al., 2018). Overall, the above data or theories support the ability of microglia to induce synaptic plasticity in neurons within the dorsal horn of the spinal cord and to maintain NP.

### 5.2 Crosstalk between astrocytes and neurons induces hyperalgesia

The activated microglia and astrocytes release various pro‐inflammatory factors and chemokines. Among them, CXCL1 is an important chemokine involved in the interaction between astrocytes and neurons. The chemokine CXCL1, also known as keratinocyte‐derived chemokines (KC), is a member of the CXC family (Ni et al., 2019). NF‐κB is involved in the upregulation of CXCL1 in astrocytes induced by TNF‐α (Xu et al., 2014). TNF‐α can stimulate the phosphorylation of astrocytes to upregulate the expression of JNK and increase the expression of connexin 43 (CX43). At the same time, JNK inhibitors and CX43 inhibitors can significantly block the expression of CXCL1 induced by TNF‐α; Therefore, the upregulated expression of CXCL1 in astrocytes is mainly regulated by the JNK signaling pathway or CX43 (Chen et al., 2014; Zhang et al., 2013). In the spinal nerve ligation model, the expression of CXCL1 and its receptor CXCR2 in the injured spinal cord astrocytes and neurons increased, respectively (Li et al., 2019). CXCL1 can directly act on CXCR2 in peripheral neurons to trigger an increase in calcium influx and indirectly induce leukocyte recruitment, thereby sensitizing primary neurons (Silva et al., 2017). The upregulation of neuron CXCR2 amplifies neuronal excitability, leading to long‐term CS, and a continuous increase in excitatory postsynaptic currents (EPSCs) in the pain circuit (Ji et al., 2019). Intrathecal injection of anti‐CXCL1 antibody can significantly reduce the pain hypersensitivity caused by spinal nerve ligation, and CXCR2 antagonists can block the thermal hyperalgesia caused by CXCL1, indicating that chemokines are involved in the NP process of CXCL1/CXCR2. In addition, chemokines to CXCL13/CXCR5 are also involved in the process of hyperalgesia. Intravenous injection of CXCL13 can stimulate spinal astrocytes to induce mechanical allodynia in mice (Jiang et al., 2016). In the spinal cord injury model (SNL), it was found that the expression of CXCR5 gene and protein in spinal astrocytes increased, and spinal cord neurons overexpressed the gene and protein of chemokine CXCL13; the expression of CXCL13 was knocked down by shRNA lentivirus transfection, hyperalgesia in SNL mice can be effectively improved (Jiang et al., 2016). See Figure 2 for details.

Glial glutamate transporter‐1 is abundantly expressed in astrocytes, which contributes to glutamate clearance from the synaptic gap and extracellular gaps (Tawfik et al., 2006). The altered expression and function of glutamate transporters can regulate glutamatergic transport and neuronal plasticity, such as LTP (Gao & Ji, 2010). TNF‐α can regulate synaptic activity through a direct action of TNFR1 on neurons (Beattie et al., 2002). TNF‐α secretion by astrocytes increases AMPA receptors and decreases GABA_A_ receptor surface expression in hippocampal neurons, resulting in increased frequency and amplitude of mini EPSCs and decreased the amplitude of mini inhibitory postsynaptic currents (Wigerblad et al., 2017). In fact, different concentrations of TNF‐α have a bidirectional effect on LTP induction, with a high concentrations of TNF‐α (1 μg/ml) having been shown to weaken LTP, whereas low concentrations (1 ng/ml) promote LTP (Maggio & Vlachos, 2018). TNF‐α in the cerebrospinal fluid of patients with chronic pain is only a few picograms per milliliter or even lower (Backonja et al., 2008), whereas the concentration of TNF‐α in serum is about 50 pg/ml (Koch et al., 2007). As these concentrations are well below the levels at which TNF‐α was found to impair LTP, TNF‐α release from reactive astrocytes may be more likely to promote synaptic enhancement (Tang et al., 2021). Similarly, in animal experiments, it can be demonstrated that astrocytes crosstalk with neurons via TNF‐α leads to enhanced synaptic transmission in the ACC and amygdala in the brain (Chen et al., 2013).

### 5.3 Crosstalk between microglia and astrocytes

In the process of spinal pain sensitization, activated microglia can regulate the innate immune function of astrocytes (Jha et al., 2019). As mentioned before, cytokines such as TNF‐α and NF‐κB secreted by activated microglia can activate astrocytes through related receptors on astrocytes (Rothhammer et al., 2018). At the same time, activated microglia can regulate the polarization of astrocytes and determine the different polarization types of astrocytes (Shinozaki et al., 2017). IL‐1α, IL‐1β, and TNF‐α secreted by M1 type microglia can directly induce astrocytes to polarize to type A1 in vivo and in vitro experiments, which helps to further release inflammatory factors and enhance M1 polarization (Liddelow et al., 2017; Tschoe et al., 2020). Activated M2 type microglia produce the anti‐inflammatory cytokine IL‐10, which matches the IL‐10 receptor mainly expressed in type A2 astrocytes, thereby promoting the secretion of TGF‐β from type A2 astrocytes and cardiotrophin like cytokine (CLCF1), thereby reducing the activation of microglia (Norden et al., 2014). The crosstalk between M2 type microglia and A2 type astrocytes can promote the survival and repair of neurons, even magnified by the unique anatomical structure of astrocytes (Ho et al., 2020). Recently, some research pointed out that the interaction between the highly expressed C3 in type A1 astrocytes and the C3a receptor of microglia regulates the C3/C3a–C3aR signaling pathway, thereby dynamically regulating the synapses of microglia to neurons, in which the pruning process induces the polarization of microglia into M1 type microglia (Lian et al., 2016). Before the discovery of glial cells and, in particular, astrocytes regulating synaptic transmission, it was thought that the fine‐tuning of neuronal activity was entirely under neuronal control (Nedergaard, 1994). Expression of multiple membrane receptors for neurotransmitters allows microglia to form crosstalk with astrocytes to regulate neuronal activity and synaptic transmission (Jha et al., 2019). Astrocytes are targets of microglial secretions and influence neuronal plasticity in a pathological context (Oberheim et al., 2012), ultimately inducing CS. Neuroinflammation‐driven microglia activation affects astrocyte connexin (CX43) hemichannel‐mediated function and thus the synaptic activity of neurons in an ex vivo model (Abudara et al., 2015). See Figure 1 for details.

## 6 RESULTS AND PROSPECTS

The causes of NP are complex and diverse. The neuroimmune interaction of the entire nervous system can cause abnormal sensory signals in the periphery, spinal cord (dorsal horn), and brain (thalamus and cortex), resulting in significant pain (Alles & Smith, 2018). The increase of inflammatory mediators induces afferent nerve sensitization by changing the expression of voltage‐gated sodium, calcium, and potassium channels, leading to spontaneous pain (Abdulla & Smith, 2001; Waxman et al., 1999).

In recent years, with the in‐depth study of NP, it has been found that in addition to glial cell activation, neurons can also maintain the continuous activation of glial cells through related chemokines and pathways. Neuron‐glial cell interaction is related to the pathogenesis of NP. The activation of microglia and astrocytes in the spinal cord leads to the upregulation of chemokines and the increase of signals between neurons and glial cells (Zhuang et al., 2007). After nerve and tissue damage in spinal cord injury, chemokines make peripheral and central nerves sensitive (Ji et al., 2014). Abnormal glial cell activity may trigger CNS sensitization through a variety of mechanisms, including the synthesis and release of neurotrophic factors (such as BDNF) and pro‐inflammatory cytokines (such as IL‐1β, IL‐6, and TNF‐α), which in turn induce long‐term growth (Viswanath et al., 2020), leading to increased excitability of neurons (Gui et al., 2020), and ultimately leading to pain sensitization (Viswanath et al., 2020).

It is worth noting that the immune system is also involved in NP. Neutrophils infiltrate into the site of acute nerve injury and release nerve growth factor and chemokine. Chemokines containing C–X–C and C–C sequences recruit macrophages and monocytes from peripheral blood through CCR1, 2 and 5 to participate in the production of pain (Murray et al., 2021). After the activation of neurons, the upregulation of immune cell function genes in DRG can further promote the recruitment of macrophages and T cells and then secrete inflammatory factors to stimulate the continued activation of microglia (Malcangio, 2019; Murray et al., 2021). In humans, the upregulation of TSPO (translocator protein) is often accompanied by the activation of glial cells. PET technology can also observe that sensory neurons in NP can regulate the activities of immune cells after stimulating a variety of receptors (Grace et al., 2021). Some literatures show that peripheral nerve injury not only causes the activation of spinal cord microglia, but also causes the activation of brain microglia, and participates in the development of affective disorder (Albrecht et al., 2021). It has also been found in humans that NP can induce negative emotions (Barcelon et al., 2019).

Future research needs to expand our understanding of the meaning of crosstalk between neurons, microglia and astrocytes in the CNS, improve the pathways and links of related crosstalk, and discover potential crosstalk targets. Finding the right entry point and finding the key receptor in the crosstalk link can effectively intervene in the crosstalk and inhibit the process of hyperalgesia. The development of receptor antagonists that have CNS permeability and can target multiple chemokine receptors may be more conducive to the treatment of NP.

## CONFLICT OF INTEREST

The authors declare that they have no conflict of interest.

### PEER REVIEW

The peer review history for this article is available at https://publons.com/publon/10.1002/brb3.2868.

## Data Availability

Data sharing are not applicable to this article as no new data were created or analyzed in this study.
